# Navigating sexual challenges in couples post breast cancer treatment: a qualitative study

**DOI:** 10.3389/fpsyg.2026.1789758

**Published:** 2026-04-09

**Authors:** Sonia Vennerød Azmi, Kristine Stormyr, Vigdis Moen, Sofie Dalheim Søgaard, Monica Jernberg Engstrøm, Randi Johansen Reidunsdatter

**Affiliations:** 1 Faculty of Medicine and Health Sciences, Norwegian University of Science and Technology, Trondheim, Norway; 2 Department of Public Health and Nursing, Faculty of Medicine and Health Sciences, Norwegian University of Science and Technology, Trondheim, Norway; 3 Department of Breast- and Endocrine Surgery, St. Olavs University Hospital, Trondheim, Norway; 4 Department of Clinical and Molecular Medicine, Faculty of Medicine and Health Sciences, Norwegian University of Science and Technology, Trondheim, Norway; 5 Department of Circulation and Medical Imaging, Faculty of Medicine and Health Sciences, Norwegian University of Science and Technology, Trondheim, Norway

**Keywords:** breast cancer, breast cancer survivor, Dyad, follow-up care, late-effects, partners, relationship, sexual challenges

## Abstract

**Purpose:**

Breast cancer (BC) treatment affects BC survivors' sexual health, causing relational challenges. Sexual health is an under-communicated topic in cancer survivorship, and partner perspectives are often neglected. This qualitative study explored (1) how sexual health is affected by BC diagnosis and treatment, and (2) how couples navigate the resulting sexual and relational challenges.

**Method:**

Semi-structured interviews were performed separately of 37 BC survivors and 19 of their partners. The study included 19 heterosexual couples and 18 BC survivors who either did not have a partner or whose partner did not participate. The participants were Norwegians aged 28 to 67 years. Thematic content analysis was carried out using systematic text condensation.

**Results:**

BC survivors experienced side effects post treatment that affected sexual health, particularly reduced sexual desire, fatigue, bodily pain, vaginal dryness and body-image concerns. Both BC survivors and partners lacked guidance regarding sexual health during BC follow-up. As a result, they developed positive and negative strategies to address their sexual and relational concerns. Positive strategies, such as redefining intimacy through alternative sexual approaches, prioritizing open communication, showing love and support, seeking help, accepting the present and maintaining hope for improvement helped couples navigate challenges constructively. In contrast, negative strategies, including skepticism toward new sexual approaches, avoiding intimacy, lack of open dialogue and feelings of guilt and rejection, often created barriers and hindered intimacy.

**Conclusion:**

Our findings highlight a significant gap in sexual health support for BC survivors in Norway, with partners often overlooked in the process. Integrating sexual health as a routine part of survivorship care through dedicated roles, clear guidelines, and consistent, timely conversations can help bridge this gap. Additionally, this study provides suggestions for topics and strategies that healthcare professionals can discuss with BC survivors and their partners as part of follow-up care. Future work should include more diverse survivor groups, evaluate implementation of stepped-care models in real-world settings, and develop multimodal interventions that combine biological, behavioral, and psychosocial components. Comparative studies across cancer types are needed to inform flexible and scalable models of sexual-health support.

## Introduction

Breast cancer (BC) is the most common cancer type among women with a lifetime risk of 11% up to the age of 80 (The Norwegian Directorate of Health, 2023). In Norway, survival rates are high, with ~93% of women diagnosed between 2018 and 2022 surviving beyond 5 years (Cancer Registry of Norway, 2022). As treatment-advances increase the number of long-term survivors, greater attention must be given toward survivorship issues. As survivorship perspectives expand, sexual health has emerged as one of the most consistently reported yet least addressed late effects of BC treatment (Hernández-Blanquisett et al., 2022; Jing et al., 2019; Franzoi et al., 2024).

Recent evidence underscores both the high prevalence and the enduring impact of sexual concerns after BC: large cohort and review papers report that ~60–75% of BC survivors experience sexual difficulties (Hernández-Blanquisett et al., 2022; Jing et al., 2019), and up to 78% report at least one sexual concern within four years after diagnosis (Franzoi et al., 2024). This corresponds to a 3.5-fold increased risk compared to the general population (Sousa Rodrigues Guedes et al., 2022), and aligns with broader evidence showing that BC survivors consistently report poorer sexual health than the general population (Åsberg et al., 2023a, 2025). Sexual challenges are closely linked to depression, reduced quality of life and lower treatment adherence in this population (Franzoi et al., 2024). These findings mirror broader oncology data showing that sexual dysfunction remains one of the most distressing and persistent survivorship issues across cancers, reinforcing the clinical relevance of systematically addressing sexual well-being in BC follow-up (Hernández-Blanquisett et al., 2022; Franzoi et al., 2024; Cucciniello et al., 2024; Agrawal et al., 2025; Dahouri et al., 2026).

Sexual health is broadly defined by the WHO as a multidimensional aspect of wellbeing that includes physical, emotional, relational, and social components (WHO, 2000). Given the complexity of sexual health, this study focuses on “sexual challenges,” which we define as difficulties affecting sexual wellbeing, including reduced desire, arousal problems, pain, difficulty achieving orgasm, and body image concerns. These challenges are best understood within a biopsychosocial framework, shaped by the interplay of biological, psychological, and social factors (WHO, 2000; Castillo et al., 2022). When thinking holistically about sexual health, one must naturally also consider the relational dimension: sexuality rarely concerns the individual alone, but unfolds between two people within a partnership. Consequently, sexual challenges affect not only survivors but also their partners, influencing the couple as a dyad. While there is growing attention to how sexual health concerns affect not only survivors but also their partners (Takahashi and Kai, 2005; Ussher et al., 2012; Shaffer et al., 2021; Canzona et al., 2019; Valente et al., 2021), the partner's perspective remains largely unexplored (Ussher et al., 2012; Chang et al., 2019).

In Norway, most BC patients receive a combination of surgery and adjuvant therapy, following national treatment guidelines that are updated at least twice per year. Surgery for BC consists of either breast-conserving surgery or mastectomy. In 2024, ~90% of early-stage cases were managed with breast-conserving surgery. Postoperative radiotherapy is often recommended after breast-conserving surgery. Neoadjuvant chemotherapy is often administered to patients with HER2-positive or triple-negative tumors larger than 2 cm. Patients with hormone receptor–positive disease may be offered endocrine therapy for 5–10 years to reduce cancer recurrence risk, although not all patients require it. Among those who are candidates and are postmenopausal, aromatase inhibitors are chosen in the vast majority of cases, whereas tamoxifen and other regimens are used far less frequently (Folkehelseinstituttet, 2025). Tamoxifen is typically the preferred option for premenopausal women, and is generally associated with more vasomotor symptoms, mood changes, and sleep disturbances compared to aromatase inhibitors, whose side-effect profile is instead dominated by joint and muscle stiffness (Folkehelseinstituttet, 2025; Hadji, 2008). Denmark and Sweden follow nearly identical national BC guidelines (NKBC, 2025; DBC Group, 2025), making these patterns representative of the broader Scandinavian landscape.

BC treatment affects sexual health through physical and psychological pathways. Physically, treatment modalities of BC such as surgery, chemotherapy, radiotherapy or endocrine therapy can all impair sexual health. For instance, endocrine therapy and chemotherapy are linked to vaginal dryness, pain and reduced desire (Hernández-Blanquisett et al., 2022; Kwan and Chlebowski, 2009), while mastectomy has been linked to poorer sexual outcomes than breast-conserving surgery (Åsberg et al., 2023a; Archangelo et al., 2019). The cumulative effects of BC treatments often lead to fatigue (Abrahams et al., 2016), which may further compromise sexual health. Psychologically, BC treatment may increase depression, anxiety, and body image concerns, all associated with poorer sexual health (Sousa Rodrigues Guedes et al., 2022; Oberguggenberger et al., 2017; Izci et al., 2016). Many survivors report a diminished sense of femininity and attractiveness, often worrying about their partner's perceptions of them (Åsberg et al., 2023a; Castillo et al., 2022; Takahashi and Kai, 2005; Canzona et al., 2019). Some studies suggest that BC can be particularly traumatic for younger women due to aggressive treatment and consequences of early menopause, while older women may cope better due to more life experience (Chang et al., 2019; Kedde et al., 2013). Similarly, younger partners may experience the diagnosis as more distressing than older partners (Rowland and Metcalfe, 2014).

Access to information, resources, and support for sexual health is a recognized human right (Starrs et al., 2018), yet sexual health remains insufficiently addressed in survivorship care (Pimsen et al., 2024). Research shows that public health care providers often fail to address sexual health during and after BC treatment due to insufficient training, lack of time, discomfort discussing sexuality and the belief that sexuality is not a significant issue in cancer care (Takahashi and Kai, 2005; Pimsen et al., 2024; Dai et al., 2020; Halley et al., 2014). Survivors are reluctant to initiate conversation with health care providers (Cucciniello et al., 2024; Takahashi and Kai, 2005), and when brought up, sexual challenges are often reduced to only its physical aspects, neglecting its multidimensional and psychosocial nature (Dai et al., 2020). Couples also struggle to communicate about sexual challenges with each other (Tat et al., 2018). There are currently no standardized strategies to address sexual challenges after BC, but evidence suggests that a couple-based and multidisciplinary approach, combining pharmacological, psychological and lifestyle strategies may improve both relationship quality and sexual health (Ussher et al., 2012; Tat et al., 2018).

### Aims of the study

Sexual health is an important factor in quality of life after BC, yet it remains insufficiently addressed in survivorship care. While existing research has established that BC survivors experience poor sexual health, there is still limited knowledge about the specific nature of these challenges and how they also affect partners and the couple as a dyad. While a few qualitative studies have explored how couples manage sexual challenges (Takahashi and Kai, 2005; Ussher et al., 2012; Shaffer et al., 2021; Canzona et al., 2019), none have been conducted in a Scandinavian context. To help bridge these gaps, this study aimed to: (1) identify the sexual challenges experienced by BC survivors after treatment and (2) explore how couples navigate the sexual and relational challenges that arise after BC. By exploring these aims, we hope to identify gaps in follow-up care after BC and improve sexual health support by highlighting the physical, psychological, emotional and relational aspects that affect both survivors and their partners.

## Methods

We used a qualitative study design with a phenomenological approach. The methods are described in line with the Standards for Reporting Qualitative Research (SRQR) guidelines (O'Brien et al., 2014), and the SRQR checklist is provided in Supplementary Table A.1.

### Participants

Eligible participants were BC survivors who had completed primary curative treatment and were no longer in active cancer therapy. Survivors receiving long-term endocrine therapy were eligible, as such treatment is common in survivorship and may influence sexual health. Our aim was to include participants who were beyond acute treatment effects and had returned to a stable everyday life, with enough time and capacity to reflect on sexuality as part of their post-treatment experience.

The recruitment was carried out in two steps:

- First, study information was provided to BC patients at the 2-year follow-up at the oncological and surgery outpatient clinic.
- Second, when the COVID-19 pandemic disrupted outpatient recruitment, we expanded to national digital recruitment. Advertisements were posted on the intranet and Facebook pages of NTNU, the Norwegian BC Association, and the National Network for BC Research. Study information was also shared on the research group members' personal social media pages.

Informed consent was provided electronically through the NTNU's MachForm solution, and participants were consecutively approached and appointments for digital interviews were made. Participants were recruited from all parts of Norway.

### Interviews

Interview guides were developed by the last author in close cooperation with the multidisciplinary research team, including user representatives. The interviews were semi-structured and covered topics such as general health, BC treatment, sexual relationship, individual sexuality, and informational needs.

All interviews were conducted by the same researcher, a registered nurse and trained sexologist with extensive experience in discussing sensitive topics related to sexuality and couple dynamics. This ensured a consistent interview approach across participants while maintaining a professional, non-judgmental stance.

BC survivors and partners were interviewed separately. At the beginning of each interview, participants were informed that nothing they shared would be disclosed to their partner. This procedure safeguarded privacy within dyads and reduced concerns about intra-couple disclosure. The couples did not have access to each other's interviews.

Most interviews were conducted digitally via audio-video Zoom meetings and lasted between 30 and 90 min. The resulting video files were stored locally on a password-protected NTNU computer, with access restricted to ethics-approved members of the research team. All audio-visual files were deleted after verbatim transcription to further minimize the risk of re-identification.

The interviews were transcribed and de-identified before analysis. Participants were assigned numerical ID codes; for dyads, survivors and partners shared the same number to enable analytic comparison while preserving confidentiality. No identifiable information was retained in the analytic dataset.

The interviewer followed the semi-structured guide consistently and applied reflexive practices to reduce potential interviewer bias (e.g., neutral prompts, avoiding leading questions, and documenting contextual reflections after each interview). These procedures were undertaken in accordance with the study's ethical approval (REK 2020/58888).

### Analysis

We employed systematic text condensation (Malterud, 2012, 2001) to analyze the transcripts. This method consists of four steps, illustrated in Supplementary Table A.2. Coding issues and uncertainties during analysis were resolved through discussion with the research group. The quotes from the interviews were translated from Norwegian to English after the analysis was completed. The translation aimed to preserve the essence of the quotes rather than providing a word-for-word rendering. Words enclosed in brackets were not explicitly articulated by the participants but added to convey the intended meaning and context accurately. The interviewer approved the translated quotes.

The data were analyzed separately for each study aim. For Aim 1, the transcripts of all 37 BC survivors were analyzed to ensure a broad representation of individual experiences related to sexual health after BC. For Aim 2, only the transcripts from the 19 BC survivors and their corresponding partners were analyzed to capture the dyadic perspectives on navigating sexual challenges after BC.

## Results

### Participant characteristics

This study included a total of 37 BC survivors. Of these, 19 participated as part of a couple, alongside their male partners. The remaining 18 BC survivors did not have a partner who participated in the study, either because they were single, or their partner chose not to take part. For clarity, we refer to this group as “*unpartnered BC survivors”* in this study. Among these unpartnered participants, three experienced a breakup with their partner during cancer treatment. Participant characteristics of all BC survivors, presented separately by partnership status, and the male partners, are shown in Table 1.

**Table 1:** Characteristics of breast cancer survivors and their partners (*N* = 37).

Age (years)	Partnered BC survivors (*n* = 19)	Partners	Unpartnered BC survivors (*n* = 18)
50–67	6	7	11
35–49	10	8	6
28–34	3	4	1
Type of work			
Health personnel	5	2	4
Other	14	17	14
Parental status			
Children living at home	10	10	8
Adult children or no children	9	9	9
Unmentioned	-	-	1
Work status			
Working	11	17	6
Retired	1	1	0
Sick leave^*^	7	0	12
Unmentioned		1	
Relationship status			
Long-term^**^	16	16	12
Recent^***^	3	3	0
Not in a relationship/Unmentioned	-	-	6
BC treatment			
Breast-conserving surgery	12	-	9
Mastectomy	7	-	9
Chemotherapy	14	-	13
Radiation therapy	17	-	12
Tamoxifen	11	-	5
Aromatase inhibitors	4	-	8
Switched between endocrine treatments	3	-	1
GnRH-agonist	3	-	3

^*^Participants reported sick leave ranging from 20% to 100% at the time of the interviews.

^**^“Long-term” = Relationship duration >2 years at time of interview.

^***^“Recent” = Relationship duration ≤ 2 years.

### Sexual challenges after BC treatment

To address Aim 1 of the study, which is to identify the sexual challenges experienced by BC survivors after treatment, we present the main sexual challenges reported by the participants. While most BC survivors had enjoyed satisfying sexual relationships prior to their diagnosis, our analysis revealed that they experienced new sexual challenges following treatment, which we have grouped into the following three themes: (1) reduced sexual desire, (2) vaginal symptoms and bodily pain, and (3) body image concerns.

**Theme 1: Reduced sexual desire**

Reduced sexual desire was the most frequently mentioned challenge in this study. It was often attributed to hormone therapy, chemotherapy and radiation. Fatigue and bodily pain further limited sexual drive, especially during or after intensive treatment phases.

“*Hormone medication drained my desire” -* BC survivor 3.

“*There was not much energy left for intimacy when you spent a week just trying to survive the fatigue and pain*.” - BC survivor 26.

“*It was hard to find a partner, as I no longer felt any chemical attraction.” -* BC survivor 32

The decline in sexual desire affected BC survivors' willingness to seek a new partner and influenced how they perceived their existing relationships. For many, sexuality was no longer instinctive but something strenuous that required deliberate effort.

**Theme 2: Vaginal symptoms and bodily pain**

Vaginal dryness and pain were widely reported issues. Attempts to manage the condition through lubricants and creams were often only partially effective. Moreover, symptoms were not limited to the vaginal area: pain in the breasts, joints, and muscles also contributed to making sexual activity uncomfortable.

“*It feels like everything down there is dry and sore” -* BC survivor 33

“*It was hard to find joy or relaxation in intimate moments when I was constantly in pain” -* BC survivor 27

Couples were unsure about how to approach intimacy without causing discomfort. Fear of pain led to some BC survivors avoiding sex altogether, which further strained relationships.

**Theme 3: Body image concerns**

Beyond pain and fatigue, many survivors struggled with changes to their appearance. Scars, altered breast shape, weight gain and hair loss challenged survivors' sense of femininity and identity and made it difficult to feel confident or desirable. The changes to the breasts were particularly difficult to cope with not only for aesthetic reasons, but because they reminded survivors of their diagnosis every time they undressed. Similarly, some BC survivors shared that hair loss was the hardest physical change to cope with because it made their illness visible to everyone. It seemed like for many, being perceived as ill was harder to manage than the actual physical changes themselves.

“*Every time I got up and moved, I could feel the prosthesis loosen its grip on my skin and hang awkwardly in my bra. And then, of course, I started thinking about cancer. The same thing happened every evening when I took it off. Cancer again. That word was so dominant in my life.” -* BC survivor 4.

“*I keep wondering how I can trust that he really wants me. I tell myself he's lying, because how could anyone want “this”? Deep down I know he does, yet somehow, I still can't let myself believe it.” -* BC survivor 6.

### Navigating sexual and relational challenges after BC treatment

In line with Aim 2, which seeks to explore how couples navigate the sexual and relational challenges that arise after BC, we present the strategies couples used to cope with the sexual side effects of BC treatment. Most couples found that regular cancer follow-up care did not provide enough information about sexual health, forcing them to tackle their sexual issues on their own. Our investigation revealed several strategies that helped couples manage these challenges, which we have grouped into the five following themes: (1) Practical sexual adjustments, (2) Communication, (3) Expressing love and support, (4) Seeking help for sexual health, and (5) Accepting the present and maintaining hope for the future.

Strategies were categorized as either positive or negative based on the perspectives of both the interviewees and analysts. Positive strategies facilitated couples to navigate challenges constructively, while negative strategies often created barriers and hindered intimacy. The terms positive and negative are used analytically and are not intended to place a moral judgment on participants' actions or experiences. Detailed summaries of themes, sub-themes and direct quotations from the interviews are presented in tables at the end of each section for deeper understanding of the reported results.

**Theme 1: Practical sexual adjustments**

The couples had to re-explore how they practiced their sexual lives to adapt to the sexual challenges following treatment. They placed greater emphasis on foreplay, oral sex, and the use of vibrators and lubricants. BC survivors would also decide the pace and duration of sex to ensure less pain. Partners were more cautious with the operated breast, but BC survivors still appreciated having both breasts engaged during sex. Some couples struggled to adjust, avoided sex altogether or hesitated to use aids.

Individual sex life was also important to adapt to their new sexual lives. Male partners masturbated frequently to meet unmet needs, while BC survivors masturbated less due to lack of sexual desire. Opinions on masturbation varied among BC survivors: some felt it enhanced intimacy, motivating them to masturbate more, while others believed it drained their already limited sexual desire.

Initiating sex became challenging due to lack of desire. Partners often initiated and experienced several rejections, which grew tiresome over time and could make them feel unwanted. Some BC survivors occasionally initiated sex, often driven by feelings of guilt toward their partner or fear of potential infidelity. To address this challenge, some couples scheduled regular sex, which helped reduce misunderstandings and eased the fear of rejection. Many found that regular intimacy strengthened their relationship and helped maintain desire.

The practical sexual adjustments the couples made, along with direct participant quotations, are presented in Table 2.

**Table 2:** Practical sexual adjustments; subthemes and quotations.

Positive strategies	Negative strategies
**Redefining sex: lubrication, increasing foreplay, oral sex, vibrators and other sexual aids, shortened intercourse, caution around operated breast while not neglecting it:** • BC survivor 9: *We must use a quarter liter of lubricant each time to avoid pain*. • Partner 15: *It's unacceptable if sex causes her pain, and that I am the one contributing to it. [...] it is important that she does not develop negative associations with sex. [...] When it's dry and uncomfortable at first, it's very important that things go at her pace*. newline **Scheduling sex to reduce misunderstandings and ease the fear of rejection. Having regular sex to strengthen the relationship and maintain desire:** • BC survivor 18: *There are always three entities in a relationship. It's he, she, and the couple. When I say I want sex, I sometimes say it's for the couple. It is for our mutual well-being, not because I feel like it, but because I know it's what's good for the couple*. newline **Maintaining an individual sex life. Masturbating to address unmet needs or enhance desire:** • BC survivor 8: *[Masturbation] is not quite identical [to sex], but it's worth a try... if I do it often myself, then it can improve our sexual life as well*.	**Not open or skeptical to new approaches, afraid or unfamiliar with sexual aids:** • Partner 11: *A sexologist cannot tell you that you will regain your desire when it's the medication that decreases it*. newline **Limiting masturbation out of fear that it might deplete libido:** • BC survivor 18: *It might be wrong, but I've never believed that the more sex you have, the more you want. I've always thought desire was a limited resource*. newline **BC survivors initiating sex out of guilt or fear of infidelity:** • BC survivor 1: *The desire was completely gone, so I only did it when I thought that enough time had passed. I think he deserves it. My husband has been very patient*. • BC survivor 9: *I felt obliged to. I was afraid that he would get it another place if he didn't get what he needed with me. Of course, I also don't want him to be sad or feel less attractive*. newline **Partners ceasing to initiate sex due to fear of rejection:** • Partner 1: *I feel that it becomes a vicious circle, because I get a rejection, upon rejection, upon rejection, and therefore I make less, and less, and less of an effort. [...] It is tiring and hard to try, and try, and put in a lot of effort, and still, you get disappointed every time*. newline Being too cautious of the operated breast, making the BC survivor feel bad hspace*-6ptor lose desire • BC survivor 1: “*I have no sensation in the breast at all. It's just skin now, and I don't feel anything there. Before, when we had sex, he would touch both breasts, but now I notice he avoids this one. It feels strange that he only touches the other breast.”*

**Theme 2: Communication**

The BC survivors emphasized the importance of engaging in open and honest conversations with their partner around their sexual challenges and new preferences. Both partners and BC survivors found that talking freely was more beneficial than ruminating on thoughts alone, which could create feelings of emotional distance, uncertainty, or resentment within the couple.

Couples also highlighted the importance of actively listening to one another and being empathetic during conversations. Without mutual understanding, open conversations could feel useless as they failed to lead to change.

Lastly, couples reported that taking time to discuss their relational issues had a positive impact on their relationship, while many others often neglected this over everyday life tasks.

We did not observe any clear patterns suggesting that couples in long-term relationships communicated better than those in shorter relationships.

For direct participant quotations and subthemes related to communication, refer to Table 3.

**Table 3:** Communication; subthemes and quotations.

Positive strategies	Negative strategies
**Open and honest conversations:** • Partner 14: *We have been talking openly […] about becoming menopausal, what treatment can do to the pubic region, a vagina that gets drier and those things. […] Yeah, so we have been talking about the fact that when I have more knowledge, it is easier to show understanding for why things are as they are*. newline **Expressing empathy and listening to one another:** • BC survivor 13: *I feel that we can say things as they are. What we have been talking about the most is probably that we have different needs. We try not to accuse each other but rather talk about what we can do to make it better and what we miss*. newline **Setting aside time for difficult conversations:** • Partner 4: *We were very conscious that we needed to [take time to] sit down and talk about things*.	**Avoiding honesty leading to uncertainty or resentment:** • BC survivor 10: *I have been afraid to let him know that [sex] is painful, because I don't want him to be afraid of touching me*. • Partner 17: *Yeah, the first natural thought is, is it me? Is it something I have done, is it... Do I not shave often enough? Do I smell bad? Is it something I have done today? Is it something you are thinking about? Like the thoughts spin a little bit. Now that I know that it's not just that she doesn't want to, but it's the medication that has an influence here. It eases my mind because I understand it better*. newline **Not trying to see your partner's point of view:** • BC survivor 18: *We have talked a lot about it, but I don't understand him. I am envious and think that it would have been so wonderful to have so much desire. He doesn't understand me, and why I don't find it more interesting*. • Partner 19: *It has kind of created a distance. That we are, like, on different planets*. newline **Letting everyday life get in the way of talking about sexual challenges:** • BC survivor 4: *There are so many things in life that need to fall into place to have a normal everyday life. It is, like, work, being a good mother, making dinner, and fixing things […], then you prioritize your sexual life far down*.

**Theme 3: Showing love and support**

Many participants emphasized the importance of maintaining intimacy when sex was challenging. Couples sought alternatives to sex like hugging, kissing, and spending quality time together, believing these acts helped sustain their relationships when sex was not as available. Some BC survivors struggled with closeness, fearing that physical intimacy might be misinterpreted as an invitation to sex. Non-sexual intimacy came easily for some but required effort from others.

While non-sexual gestures were helpful, having sex remained important for many couples. Without it, they felt more like friends than lovers.

Having a supportive partner was helpful while experiencing sexual challenges. Many women experienced an increase in sexual desire when their partner showed understanding and appreciation. Partners offered support through compliments, patience, acceptance, and by helping with household chores and children. However, juggling multiple responsibilities while caring for the survivors could become exhausting, leaving some partners struggling to address their own needs.

Direct participant quotes regarding the sub-themes that were just presented are presented in Table 4.

**Table 4:** Showing love and support; subthemes and quotations.

Positive strategies	Negative strategies
**Non-sexual intimacy: Finding ways to be close and intimate other than sex. Spending quality time together:** • BC survivor 6: *We've forgotten a bit about being together without having sex* • BC survivor 1: *We started to just set aside time every evening for a week just for us. He dropped watching the game, I dropped my friends, and then it continued for a bit, that we were like “what are WE doing tonight?”* newline **Supporting your partner in everyday life:** • Partner 13: *I reassured her about her looks early on. It's not her fault that it turned out that way. You must accept things the way they are*. • BC survivor 10: *He shows everything he needs to show through action. […] there is a lot of love in taking the children out for a walk or taking out the rubbish and cooking dinner every day*.	**BC survivors avoiding any form of intimacy out of fear it might be misinterpreted as an invitation to sex:** • BC survivor 8: *I love cuddling and being next to him. But it becomes a bit annoying when he gets in the mood and wants us to do something more, when that wasn't my intention. In these situations, I can feel a little pressured to [have sex with him]. I initially didn't want to, but then I feel that he does, so we'll just do it either way. [...] I like to be careful about what I do so as not to give him false hopes*. newline **Caring for your partner and forgetting your own needs:** • Partner 15: *I was a nurse, cook, maid, and emotional support—every single day. It was exhausting*.

**Theme 4: Seeking help for sexual health**

Most BC survivors and partners had not been informed about the sexual side effects of treatment, or they had received the information at a time when they were not susceptible. Several couples felt alone regarding the sexual and relational challenges they faced. Most partners felt excluded from conversations about the late effects of BC treatment and desired more information, while some thought it was enough to receive information from their partner.

Some couples independently sought out information online, from professionals such as doctors or therapists, or from individuals with similar experiences. Reported barriers to seeking help included perceiving it as too burdensome, not knowing where to find appropriate support and not recognizing the connection between sexual challenges and BC treatment. One partner expressed skepticism about the effectiveness of counseling or “talking through” issues that were perceived as primarily physical, such as reduced sexual desire. A few BC survivors described feeling ashamed to “bother” healthcare personnel with their sexual concerns, believing they should instead be grateful simply to be alive. Several participants also noted that healthcare providers appeared uncomfortable when the topic of sexual health was raised or that they had little to offer in response.

Couples wanted clear advice on managing side effects, available support options, and practical strategies for improving sexual wellbeing. The participants praised professionals who introduced the topic unprompted. Survivors believed discussions about sexuality should begin early in the treatment process but be balanced to avoid overwhelming patients. One BC survivor suggested that one should have had a session with a sexual therapist once treatment was finished.

Table 5 provides further details on the barriers and strategies related to seeking help.

**Table 5:** Seeking help for sexual health; subthemes and quotations.

Positive strategies	Negative strategies
**Seeking advice from professionals: Gynecologists, oncologists, cancer coordinators, general practitioners, sex therapists, psychologists, and couples' therapists:** • BC survivor 10: *For me, I think that one thing is the physical, but much is also in my head. For me to have better sexual health I need to have a good phycological health. I need to process things. There is too little of it in the cancer follow-up*. newline **Seeking information from other cancer survivors and partners:** • BC survivor 8: *You need to get [information] from like-minded people, either if it is next of kin who gets to speak with other next of kins, or a cancer patient that gets to speak to other cancer patients*. newline **Finding information online, participation in group conversations, cancer seminars**. No single quotation captured the full meaning of this subtheme; it reflects a pattern described across several interviews.	**Barriers for seeking help for sexual health:** • BC survivor 7: *As a cancer survivor you don't have anyone to call. You are out of the system. You don't have any more follow-up appointments. You need to go to your GP, and that is too bothersome*. • BC survivor 28: *I tried to find information, but there was so little... I didn't think it should have been my job to figure these things out*. • BC survivor 21: *Healthcare professionals found it uncomfortable to talk about [sexual health]. When I asked directly... they didn't really have anything to offer*. • BC survivor 10: *Yes, I do admit that we should have had more information, practical information. How [the disease] affects you, what you can do to improve things, and what you are entitled to and not*. • BC survivor 21: *Along the way, memory, among other things, is heavily affected. Remembering the information I was given was difficult. And I believe that if I had been given information about sexuality at that time, it would simply have gone straight into the spam filter, because it was just too much. You must remember all the medications, what type of cancer you have, there are so many things*.

**Theme 5: Acceptance of the present while remaining hopeful toward the future**

Several couples explained that it was important for them to accept their altered sexual life as it was and appreciate other aspects of their relationship. Older couples often expressed acceptance of their altered sex life and explained that their sexual desire had already been decreasing as part of growing older. Most partners held a positive outlook on the sexual challenges and their significant others' bodily changes. In contrast, some BC survivors expressed fear of disappointing their partner due to body-image concerns and sexual challenges.

In addition to acceptance, many couples remained hopeful for better sexual experiences after completing endocrine therapy. Some couples believed that, with time, they would find ways to navigate these challenges together. Some participants in long-term relationships also described that they had previously overcome difficult periods together, which contributed to a belief that things would improve again with time. Although half of the participants had children living at home, parenthood did not appear to shape how couples accepted their situation or stayed connected during the challenges following BC.

See Table 6 for more participant quotations related to hope and acceptance.

**Table 6:** Acceptance and hope; subthemes and quotations.

Positive strategies	Negative strategies
**Accepting sexual life as it is:** • Partner 16: *The sexual challenges are part of the package. I love her so much, that this is something I need to deal with*. newline **Hope that sex will get better over time and after ended endocrine treatment:** • Partner 15: *It was very uplifting for me to hear [health personnel] explain to us that she will eventually stop taking the [endocrine] medication. You must be allowed to hope that if it [the sex life] doesn't become perfect, at least it will get better*. • BC survivor 9: *I have hope that it will eventually get better. I tell myself that this is just a phase. I want to be with him for the rest of my life, so hopefully we will get back [to having sex] again*. • Partner 8: *We've had some tough periods over the years for various reasons, but deep down I've always had a certain calm because I believe we'll get through it*.	**Mourning the previous sex life and fearing to disappoint your partner:** • BC survivor 12: *I am sorry that I cannot give him what one should in a relationship*. newline **Being disappointed with post-treatment body:** • BC survivor 1*: I hate that boob, it's... I don't want to see it. It is ugly, and it reminds me of everything, and I don't understand that he thinks... that it looks ok, and he says that it does, that it doesn't bother him at all, but I cannot get comfortable with it*.

## Discussion

We conducted in-depth interviews with 37 BC survivors and 19 of their partners to explore what sexual challenges arise after BC treatment and how couples have managed them.

BC survivors commonly reported sexual side effects like reduced sexual desire, vaginal dryness, pain and body-image concerns. The reduction in sexual desire and the presence of vaginal symptoms in BC survivors can be attributed to both endocrine therapy and chemotherapy, which markedly reduce circulating estrogen and androgen levels. Chemotherapy is known to induce premature ovarian insufficiency in many women, leading to abrupt hormonal changes, vaginal dryness, pain, fatigue, and reduced libido, effects that may persist long after treatment completion (Hernández-Blanquisett et al., 2022; Kwan and Chlebowski, 2009; Scavello et al., 2019). Radiation therapy has also been associated with poorer sexual outcomes, both through direct physical changes to the breast tissue and because it often reflects a more intensive overall treatment course which can contribute to long-term fatigue, altered body image, and decreased sexual functioning (Albornoz et al., 2014; Streicher and Simon, 2018). It is difficult to determine whether the symptoms experienced by our participants stemmed from BC treatment or from natural aging and menopausal transition, and our study was not designed to distinguish between these pathways. In fact, it is known that sexual functioning declines with menopause, with similar symptoms such as vaginal dryness, pain and diminished desire (Scavello et al., 2019; Paschou et al., 2024). Many of the psychosocial factors affecting BC survivors such as body-image concerns, relationship quality, coping styles, and life stressors are also established contributors to impaired sexual function in healthy menopausal women too (Scavello et al., 2019). Our study population were within the typical age range for natural menopause, and we did not systematically collect information on whether menopause had been induced by treatment or had already occurred before the cancer diagnosis. This should therefore be considered a potential confounding factor.

When describing how couples managed the challenges after BC, participants described receiving little guidance about sexual health during BC follow-up and therefore developing their own strategies, both helpful and unhelpful. Strategies the couples experienced as helpful included exploring alternative sexual approaches, scheduling sex, using sexual aids and maintaining physical and emotional closeness beyond having sex. These results align with earlier research advice to challenge the coital imperative through exploration of non-coital intimacy (Ussher et al., 2012). Educating patients and couples that sex is more than penetration is vital in this context. Additionally, having an open conversation within the couple and a strong partnership when dealing with the repercussions of BC treatment were crucial for the participants, also consistent with existing knowledge (Sousa Rodrigues Guedes et al., 2022; Ussher et al., 2012; Canzona et al., 2019; Walsh et al., 2005; Loaring, 2012). Prior evidence reveal that younger cancer survivors and those in less-established relationships can face greater relational strain which may amplify sexual health challenges (Rabin, 2019). In our interviews, some participants in long-term relationships described greater optimism that the sexual challenges would improve over time, however, we did not observe a consistent pattern indicating that long-term status was associated with better communication or more favorable sexual outcomes. Rather, communication quality itself appeared to be the most important factor regardless of relationship duration.

The couples wanted more information and support regarding sexual and relational challenges after BC, and partners often wished to be involved. These findings support previous evidence that healthcare providers often fall short in addressing sexual health concerns (Takahashi and Kai, 2005; Ussher et al., 2012; Pimsen et al., 2024; Dai et al., 2020; Halley et al., 2014; Tat et al., 2018), partly due to insufficient knowledge, misconceptions around the topic, lack of time and unclear responsibilities for initiating these conversations (Pimsen et al., 2024; Canzona et al., 2018). One potential misconception is that older adults are not sexually active or that sexuality is unimportant to them (Sinković and Towler, 2019). However, our study highlights that sexuality remains significant at all ages. In Norway, 90% of men aged 65–75 are sexually active, as are 80% of women aged 65–69 and 60% of women aged 70–75 (Sunde et al., 2024). Furthermore, having an active sexual life is regarded as important even in ages up to 79 years (Åsberg et al., 2023b). Hence, it is crucial for healthcare providers to recognize and address sexual health in older patients.

Timing is a crucial factor when addressing sexual concerns. Our findings suggest that addressing sexual health too early in cancer care may lead to information being forgotten, while waiting too long may exacerbate issues. Opinions regarding the best timing vary, but repeated discussions could be beneficial (Dai et al., 2020). Based on findings and discussions at the European Cancer Survivorship Symposium in September 2024, involving doctors, nurses, researchers, and sexologists, it was suggested that about 1 year post-primary treatment is an optimal time to address sexual health concerns in BC survivors and partners. Additionally, information about sexual health should be made available in various formats, such as brochures, online resources, workshops, or individual consultations, to ensure that patients and their partners have access in a form that suits them. In general, health personnel should inform and preferably repeat information regarding sexual health, as well as normalizing sexual challenges after cancer treatment.

There is a need to determine who should take responsibility for addressing the sexual health needs of BC patients and their partners, and to determine the level of knowledge required for healthcare providers to effectively address these concerns. Given an already overburdened healthcare system in Norway, we believe the solution is not to add more responsibilities to oncologists and surgeons but to introduce a dedicated role in the patient's treatment journey to address these concerns. Some studies indicate that BC survivors may prefer discussing sexual issues with cancer nurses of the same gender (Pimsen et al., 2024). Counseling models suited to BC survivors suggests that a non-specialized professional can be sufficient, provided they possess strong communication skills and encourage their patients to express their concerns openly (Cucciniello et al., 2024). Alternatively, general practitioners may be well-positioned to address sexual health topics, as they can provide ongoing care and may be familiar with the survivor's partner. Group counseling is another viable alternative, as highlighted in previous studies (Cucciniello et al., 2024). In our study, particularly partners appreciated connecting with like-minded individuals. Ultimately, the priority should be to establish clear guidelines and routines for consistently addressing sexual health, ensuring these concerns are not overlooked. Our study reflects this, as couples sought help from various healthcare professionals and appreciated any approach that addressed sexual health, regardless of the specific professional involved.

Several counseling models exist to guide healthcare in discussing the topic sexual health with patients. For instance, the BETTER model (Mick et al., 2004), designed for nurses, includes six steps: bring up the topic, explain that sex is a part of life, tell patients that resources are available to address their concerns, time the interventions appropriately, educate patients on the sexual side effects of treatment, and record all assessments and interventions in the medical record. The PLISSIT model (Almeida et al., 2019), on the other hand, emphasizes that professionals do not need to be experts to provide guidance. Instead, they should be able to initiate open discussions, offer basic advice, and refer patients to specialists when necessary. Beyond these models, more targeted therapeutic approaches such as couples therapy, sex therapy and cognitive behavioral therapy have also been shown to be effective in addressing sexual challenges BC (Marsh et al., 2020).

The strength of this study is its larger sample size compared to many other qualitative studies in this field. It also adds new knowledge by exploring sexual health in a broader way, not just the physical side, but also the psychological and relational aspects. Including both BC survivors and their partners offers a unique dyadic perspective on how couples experience and manage sexual health after cancer. This makes the study one of few worldwide and the first of its kind in Scandinavia to examine both perspectives within the same dyad. Participants had varied motivations for participating; some shared success stories to inspire others, while others sought to improve their sexual lives or relationship. However, certain limitations should be acknowledged.

Couples who chose to participate may have been more informed and interested in sexual health, introducing a potential selection bias. Additionally, the first and second author, who analyzed the transcripts, were not involved in the interview or transcription processes, which could affect the accuracy of the results. To mitigate this, the interviewer was consulted and approved the analysts' interpretations. Furthermore, some nuances may have been lost during translation of Norwegian quotes into English. Finally, as with most research in this field, the study focuses exclusively on cisgender women with male partners. There remains a significant gap in research addressing the sexual health experiences of more diverse populations, including men with BC and individuals from gender and sexual minorities, whose needs may differ (Kennedy et al., 2024).

## Clinical implications

Our findings, together with recent survivorship evidence, highlight that sexual health needs to be addressed routinely and proactively after BC treatment. Large longitudinal data show that about three-quarters of BC survivors report at least one sexual difficulty from diagnosis to 4-year follow-up, yet fewer than half receive any supportive care (Franzoi et al., 2024). This mismatch between burden and care access underscores the need for more systematic follow-up. In practice, this calls for:

- timely post-treatment check-ins focused on sexual health (e.g., at 6–12 months and again at 18–24 months),
- brief, validated screening tools embedded in routine follow-up (e.g., EORTC QLQ-SH22, Greimel et al., 2021) to identify concerns and treat accordingly, and
- clear care pathways combining education, symptom-directed measures (lubricants/moisturizers, pelvic floor therapy, pain management) and psychosexual or couple-based counseling (Cucciniello et al., 2024; Agrawal et al., 2025).

As partners often experience parallel concerns and informational gaps (Flechtenmacher et al., 2026), partner-inclusive encounters (when acceptable to the survivor) may improve shared understanding and adherence to strategies, consistent with emerging dyadic evidence. Making sexual-health discussions a routine part of care, assigning responsibility within the care team, and using stepped-care models (e.g., BETTER/PLISSIT) can help integrate sexual health as a core survivorship domain.

## Conclusion and future directions

This study highlights a significant gap in sexual health support for BC survivors in Norway. Despite the recognized importance of sexual wellbeing, the participants were largely left to navigate complex sexual and relational challenges on their own, often without guidance or resources. This lack of structured support can lead to both adaptive and maladaptive coping strategies, influencing quality of life and relationship satisfaction. Our findings call for a paradigm shift in survivorship care: sexual health must be integrated as a core component of rehabilitation, not treated as an optional topic. Establishing dedicated roles, clear clinical guidelines, and systematic, timely conversations about sexual health could help bridge the current gap. Importantly, this study provides actionable insights for healthcare professionals, including concrete topics and approaches for engaging both survivors and partners in care. By highlighting the dyadic perspective, this research not only fills a significant knowledge gap but also sets the stage for more inclusive and holistic cancer rehabilitation.

Future work should broaden the focus beyond cisgender women and male partners to ensure that diverse groups of survivors are represented. It will also be important to test implementation strategies that integrate brief sexual-health screening and stepped-care models into routine follow-up through pragmatic trials that assess uptake (whether clinics and patients actually use the tool), effectiveness (whether it improves sexual wellbeing), and equity (whether it works fairly across different groups of survivors). There is also a need for intervention studies that combine biological (e.g., non-hormonal local therapies where appropriate), behavioral (such as pelvic floor therapy), and psychosocial (e.g., sex or couple therapy) component, benchmarked against usual care and stratified by endocrine therapy exposure and menopausal status. Finally, research should extend beyond BC. Comparative survivorship studies across cancer types are needed to map similarities and differences in sexual concerns, recovery patterns, and supportive-care needs. Such work can help develop flexible models that can be adapted across oncology settings.

## Data Availability

The datasets presented in this article are not readily available because it contains information that could compromise the privacy of research participants and missing consent and ethical approval for public sharing of the dataset. Requests to access the datasets should be directed to Randi J. Reidunsdatter, randi.j.reidunsdatter@ntnu.no.
