# Supplementary material for: Navigating sexual challenges in couples post breast cancer treatment: a qualitative study
Source: Front Psychol. 2026 Apr 9;17:1789758. doi: 10.3389/fpsyg.2026.1789758 (PMC13102775; doi:10.3389/fpsyg.2026.1789758)
Supplement: Supplementary file 1 [file Table_1.docx]

**Supplementary Table A.1 – SRQR Checklist**

| **No.** | **Topic** | **Item** |
| --- | --- | --- |
| **Title & Abstract** | | |
| S1 | Title | Navigating Sexual Challenges in Couples Post-Breast Cancer Treatment |
| S2 | Abstract | **Purpose**: Breast cancer (BC) treatment affects BC survivors’ sexual health, causing relational challenges. Sexual health is an under-communicated topic in cancer survivorship, and partner perspectives are often neglected. This qualitative study explored (1) how sexual health is affected by BC diagnosis and treatment, and (2) how couples navigate the resulting sexual and relational challenges.  **Method**: Semi-structured interviews were performed separately of 37 BC survivors and 19 of their partners. The study included 19 heterosexual couples and 18 BC survivors who either did not have a partner or whose partner did not participate. The participants were Norwegians aged 28 to 67 years. Thematic content analysis was carried out using systematic text condensation.  **Results:** BC survivors experienced side effects post treatment that affected sexual health, particularly reduced sexual desire, fatigue, bodily pain, vaginal dryness and body-image concerns. Both BC survivors and partners lacked guidance regarding sexual health during BC follow-up. As a result, they developed positive and negative strategies to address their sexual and relational concerns. Positive strategies, such as redefining intimacy through alternative sexual approaches, prioritizing open communication, showing love and support, seeking help, accepting the present and maintaining hope for improvement helped couples navigate challenges constructively. In contrast, negative strategies, including scepticism toward new sexual approaches, avoiding intimacy, lack of open dialogue and feelings of guilt and rejection, often created barriers and hindered intimacy.  **Conclusion:** Our findings highlight a significant gap in sexual health support for BC survivors in Norway, with partners often overlooked in the process. Integrating sexual health as a routine part of survivorshiup care thorugh dedicated roles, clear guidelines, and consistent, timely conversations can help bridge this gap. Additionally, this study provides suggestions for topics and strategies that healthcare professionals can discuss with BC survivors and their partners as part of follow-up care. Future work should include more diverse survivor groups, evaluate implementation of stepped‑care models in real‑world settings, and develop multimodal interventions that combine biological, behavioural, and psychosocial components. Comparative studies across cancer types are needed to inform flexible and scalable models of sexual‑health support. |
| **Introduction** | | |
| S3 | Problem formulation | Sexual challenges are common among BC survivors. Sexual health plays an essential role in overall quality of life and well-being. However, the topic is often neglected in cancer survivorship care, and partners are seldom involved. We were interested in finding out 1) what were the main sexual challenges after BC treatment, and 2) how couples, as a dyad, manage to navigate through the sexual repercussions of BC treatment. |
| S4 | Purpose of research question | To investigate how BC survivors and partners are affected by the sexual repercussions of BC treatment and identify which strategies they use to manage the sexual and relational challenges after BC treatment. |
| **Methods** | | |
| S5 | Qualitative approach & research paradigm | To answer the research question, we employed systematic text condensation, a pragmatic approach inspired by phenomenology. |
| S6 | Researcher characteristics & reflexivity | The researchers did not know the participants. Given the qualitative nature of the study, the authors were mindful of their subjective role, which could impact both the analysis and findings. The main analysts (SVA and KS) documented their preconceptions before the analysis to remain aware of their own assumptions and biases. Additionally, we employed analyst triangulation, involving multiple analysts in coding and theme development. This collaborative approach enriched the interpretation by incorporating diverse perspectives of people from varied backgrounds. To ensure trustworthiness, we followed the SRQR checklist which includes questions that helped us critically appraise the study's methodological rigor and the clarity and validity of our findings. |
| S7 | Context | In Norway, BC survivors undergo a standard two-year follow-up at the oncological and surgery outpatient clinic. In 2020, women attending these follow-up appointments at St. Olavs University Hospital in Trondheim were provided study information. To recruit further participants, the study was promoted on NTNU’s intranet and Facebook pages, as well as through [the Norwegian BC Association](https://www.brystkreftforeningen.no/) and the [National Network for BC Research](https://breastcancerresearch.no/) From these locations study information was shared on the research group-members' personal social media pages. Informed consent was provided electronically through the NTNU’s MachForm solution, and participants were consecutively approached by telephone and appointments for interviews were made. The COVID-19 pandemic necessitated a shift to digital recruitment and interviews. Conducting digital interviews from the comfort of participants' homes fostered a relaxed and open atmosphere and allowed us to recruit diverse participants from all parts of Norway. |
| S8 | Sampling strategy | Eligible participants were BC survivors who had completed curative treatment but could still be receiving endocrine therapy. The sampling was purposive, targeting participants with recent experiences with BC who were open and willing to share their perspectives, and who also had a partner available for interviews. Our aim was to include participants who were beyond acute treatment effects and had returned to a stable everyday life, with enough time and capacity to reflect on sexuality as part of their post‑treatment experience. Sampling concluded once no additional participants wanted to enroll. |
| S9 | Ethical issues pertaining to human subjects | The study was approved by the Regional Committee for Medical Research Ethics, REK 2020/58888. In all parts of the project, participation was based on written, informed consent. |
| S10 | Data collection methods | Data was collected through semi-structured interviews. Interview guides were developed by the last author (RJR) in close cooperation with the multidisciplinary research team, including user representatives. The interviews covered topics such as general health, BC treatment, sexual relationship, individual sexuality, and informational needs. BC survivors and partners were interviewed separately by the third author (VM). The couples did not have access to each other’s interviews. Most interviews were conducted digitally via audio-video zoom-meetings, lasting between 30 and 90 minutes. Recruitment was ceased after completion of 56 interviews, in 2021. |
| S11 | Data collection instruments & technologies | The interviews were digitally recorded. Recordings of the interviews were verbally transcribed and de-identified using numbers from 1 to 38, paired individuals sharing the same number (1 to 19). This made it easier to connect and compare the experiences of the dyads during the analysis. All data has been stored securely on the last author’s (RJR) shared drive on NTNU’s servers, and identifiable data such as the participants’ names and recordings are stored in a password-protected file. Only the last author (RJR) and third author (VM) have access to participant details. The first authors (SVA and KS) have access to the audio-recordings and the de-identified transcripts. Only de-identified data is reported. |
| S12 | Units of study | There were 37 BC survivors and 19 partners who fitted the criteria for the study. Depending on the richness of the interviews, some were used more extensively in the reported results than others. |
| S13 | Data processing | The data was coded manually in Microsoft Word using a color-coding system.  Descriptive statistics were generated by systematically reviewing the interviews and creating a table of participant characteristics based on their responses. |
| S14 | Data analysis | We conducted a simple thematic analysis to identify the primary challenges reported by the BC survivors and partners regarding sexual health after treatment.  To identify the strategies couples used to address the sexual repercussions of BC treatment we employed systematic text condensation. This method consists of four steps, illustrated in **Table A.2.** All coding was done by the first authors (SVA and KS). Analysis began in the fall of 2023. Coding issues and uncertainties during analysis were resolved through discussion with all members of the research group. |
| S15 | Techniques to enhance trustworthiness | All coding and quotes were approved by the interviewer (VM). Transcripts were double coded by the first authors (SVA and KS), and a final coding was agreed upon after discussion. Each transcript was re-coded as more interviews were analysed, and new codes emerged. Regular discussions between researchers occurred regarding steps in the analysis to minimize subjectivity and make room for alternative interpretation of the material.  The quotes from the interviews were translated from Norwegian to English after the analysis was completed. The translation aimed to preserve the essence of the quotes rather than providing a word-for-word rendering. Words enclosed in brackets were not explicitly articulated by the participants but added to convey the intended meaning and context accurately. The interviewer (VM) approved the translated quotes. |
| **Results/findings** | | |
| S16 | Synthesis & interpretation | Participant characteristics are presented in **Table 1** and **Table 2.**  **Main sexual challenges after BC treatment:** Before BC, most couples enjoyed a satisfying sexual relationship, but after treatment, BC survivors faced challenges like vaginal dryness, bodily pain, diminished sexual desire and body image concerns.    **Strategies to navigate the sexual and relational repercussions of BC treatment:**  Most couples found that regular cancer follow-up care did not provide enough information from healthcare professionals about sexual health, forcing them to tackle their sexual issues on their own. Our investigation into the strategies couples employed to cope with the sexual side effects of BC treatment revealed 5 overarching themes: (1) Practical sexual adjustments, (2) Communication, (3) Expressing love and support, (4) Seeking help for sexual health, and (5) Accepting the present and maintaining hope for the future.  Strategies were categorized as either positive or negative, based on interviewee and analyst perspectives. Positive strategies facilitated couples in constructively navigating challenges, while negative strategies often created barriers and hindered intimacy. Detailed summaries of themes, sub-themes and direct quotations from the interviews are presented in tables at the end of each section for deeper understanding of the reported results. |
| S17 | Links to empiric data | The analytical texts in the results section conveys the participants' experiences in a condensed form, while staying close to the original data. Selected direct interview quotations support the identified themes and subthemes. |
| **Discussion** | | |
| S18 | Integration with prior work, implications, transferability, and contribution(s) to the field | Both BC survivors and their partners were affected by the sexual repercussions of treatment, and this study is the first in Scandinavia to include the perspectives of partners within a dyadic framework. The findings align with international literature demonstrating that sexual challenges after BC are highly prevalent, multidimensional, and often insufficiently addressed in follow‑up care. By integrating participants’ accounts with established counselling models (e.g., BETTER, PLISSIT) and evidence from oncology, psychology, and sexology, the study contextualizes how couples’ strategies reflect broader theoretical understandings of sexuality, communication, and relational adjustment after cancer.  This work advances the field by documenting a persistent gap in sexual‑health support within Norwegian BC survivorship care and illustrating how this gap affects both individuals and couples. The results have concrete clinical implications: they underscore the need for sexual health to be integrated systematically into survivorship pathways through dedicated roles, clear guidelines, routine and timely discussions, brief screening tools, and accessible care pathways that combine symptom‑directed measures with psychosocial and couple‑based interventions. These recommendations build directly on existing survivorship frameworks and contribute actionable guidance for healthcare systems seeking to address sexual health more proactively.  The study’s findings are transferable to similar healthcare settings, particularly those in publicly funded systems with comparable follow‑up structures and resource constraints. Its dyadic approach offers insights relevant across cancer types, supporting future work aimed at developing flexible models that can be adapted beyond BC. By identifying both systemic barriers and relationship‑level processes, the study contributes new conceptual and practical knowledge that can inform clinical practice, intervention development, and research prioritization in sexual‑health care for cancer survivors. |
| S19 | Limitations | A potential limitation of all qualitative analysis is bias, because the researcher is the primary analysis tool. Furthermore, as participation was voluntary, couples may have been more informed and interested in sexual health, suggesting potential selection bias. The main analysts (SVA and KS) were not involved in the interview process or transcription, which could affect the accuracy of the results, though the interviewer (VM) was consulted and approved the analysts’ interpretations. Lastly, some nuances may have been lost in the process of translating the Norwegian quotes into English. Menopause status was not collected systematically, which may be a confounding factor in the study. |
| **Other** | | |
| S20 | Conflicts of interest | The authors declare that there was no conflict of interest |
| S21 | Funding | Funding source: This research was funded by the Norwegian University of Science and Technology (NTNU) and The Norwegian Breast Cancer Association. The funder did not play a role in study design, data collection, analysis or interpretation nor were they involved in the preparation and submission of this manuscript. Neither party received direct benefits or access to identifiable information beyond published findings. |

**Supplementary Table A.2 - Performance of the 4-step analysis**

| **Steps** | *Step 1: Total impression* | *Step 2: Identifying meaning units and sorting them into code groups* | *Step 3: Condensation* | *Step 4: Synthesizing* |
| --- | --- | --- | --- | --- |
| **Performance** | The first authors (SVA, KS) individually read the transcripts, identifying recurring ideas and formulating 6-8 preliminary themes each. Preliminary themes were discussed and refined into five codes based on input from the research group and information from the interviews. Each code was assigned a color. Codes emerged throughout the analysis, and interviews were re-coded whenever new codes appeared. | Coding was performed manually in Microsoft Word using a color-coding system. Transcripts were double coded by the first authors. Some variations in coding occurred between authors, but a final coding was agreed upon after discussion. Meaning units were sorted into code groups in 5 separate documents. | The code groups were divided into 2-4 subgroups depending on how many nuances were found in each code group. The contents of each subgroup were written into condensates. “Golden quotes” were extracted from the interviews to illustrate the essence of each condensate. Due to thematic width, subgroups were illustrated by multiple quotes, which differs from Malterud’s guidelines. | Condensates were used to write analytical texts for each subgroup. The quotes were translated to English and woven into the text to enrich the content. The 5 analytical texts in the results chapter are direct products of the 5 codes obtained in step 1. The titles of the paragraphs differ slightly from those of the codes as they were changed during the writing process to better fit the final content of the analytical texts. |
| **Example** | “Measures to improve sex life” was identified as a preliminary theme. It developed into the code “Adjustments to changes in the sexual life”.  Codes obtained in step 1 in chronological order:  1) Adjustments to changes in sexual life  2) Communication and understanding  3) Showing support and love  4) Need of/Seeking professional help and guidance  5) Accepting the current situation | Meaning unit:  Partner 11: *She now experiences soreness in the vagina much more quickly, which wasn’t the case before the diagnosis. We must use lube* *whenever we have sex.*  The meaning unit above was sorted into code group 1) Adjustments to changes in sexual life | The meaning unit quoted in step 2 was sorted into the subcategory *Sexual aids*, which was later redefined as sub-theme *Redefining sex.*    The following “golden quote” was used to best illustrate the condensate:  BC survivor 9: *We must use a quarter liter of lubricant each time to avoid pain.* | The code 1) Adjustments to changes in sexual life, became the title *Practical sexual adjustments*. |
